# Editorial: HLA and KIR Diversity and Polymorphisms: Emerging Concepts

**DOI:** 10.3389/fimmu.2021.701398

**Published:** 2021-05-17

**Authors:** Martin Maiers, Ramit Mehr, Malini Raghavan, Jim Kaufman, Yoram Louzoun

**Affiliations:** ^1^ National Marrow Donor Program, Minneapolis, MN, United States; ^2^ Faculty of Life Sciences, Bar-Ilan University, Ramat Gan, Israel; ^3^ Michigan Medicine, University of Michigan, Ann Arbor, MI, United States; ^4^ Department of Pathology, University of Cambridge, Cambridge, United Kingdom; ^5^ Institute for Immunology and Infection Research, University of Edinburgh, Edinburgh, United Kingdom

**Keywords:** HLA, KIR, diversity, immunogenetics, polymorphism SIP- Society for Immune Polymorphism

Polymorphisms of immune system genes, including Human Leukocyte Antigens (HLA) and the Killer Ig-Like Receptors (KIR), offer limitless depths of complexity. Jean Dausset, who shared the Nobel Prize in 1980 for the discovery of the HLA system, when presented with new results showing the role of specific amino acid polymorphisms on the function of HLA, responded enthusiastically that “we need to go further and study HLA at the atomic level”.

The major histocompatibility complex (MHC) and KIR genetic loci are strongly associated with the outcomes of infectious diseases, cancers, inflammatory and autoimmune diseases, reproduction, and transplantation. Recent advances in characterizing HLA and KIR diversity in human populations have led to translational impacts for bone marrow and solid organ transplant matching.

There is currently robust discussion on the origins of HLA, MHC and KIR polymorphisms in humans, primates and other mammals, but limited information about the effects of the polymorphisms on interactions mediated by HLA and KIR. While both the HLA and KIR immune genes regions have been independently associated with diseases and their outcomes, the interactions between those regions has had limited attention. This Research Topic was conceived to appeal to researchers that have found their way, *via* different paths, to the crossroads of the polymorphisms and population dynamics of a *particular* immune sub-system with the recognition that, to progress in our understanding, we also need to establish a form of systems immunology to study the *interaction* of various polymorphic components.

There are established studies that focus on each of these areas: HLA, T cells, B cells, NK cells and their receptors. We are interested in the intersection: what emerges in combination at the synapses of specific molecules in the context of the entire organism or population.

The resulting Research Topic is 15 papers, 14 of which primarily focus on only one of the systems in humans and primates, and provide important insights into receptor repertoire diversity, diversification mechanisms, haplotypes, expression, sequencing, donor matching, peptide repertoires, and disease linkages among:

-**KIR and other NK receptors** (Solloch et al.; Alicata et al.; Roe, Vierra-Green et al.; Roe, Williams et al.; Roe and Kuang; Bruijnesteijn et al.; Cisneros et al.; Cubero et al.)
-**HLA/MHC** (Yamamoto et al.; Kavadichanda et al.; Nunes et al.; Kaufman **)**
**-MICA/B** (Klussmeier et al.)
**-Immunoglobulin heavy chains** (Rodriguez et al.)

Alas only one study explicitly focused on the interaction of HLA and KIR (Vargas et al.).

The studies presented in these articles have implications for understanding disease risk, outcomes and pathogenic mechanisms, host defense outcomes, gene classification and nomenclature, and transplantation. The studies also identify a gap - the need for new ways to bring together researchers in adjacent areas studying genetic polymorphism in the different branches of the immune system and to expose them to the various challenges, methods and advances in each sub-field.

To that end, we have developed a charter for a new *Society for Immune Polymorphism - SIP*. We envision this society as an international membership organization of scientists dedicated to understanding genetic and functional variations in the vertebrate immune system, and the vertebrate immune system’s role in health, disease, and evolutionary biology.

Our goal is to promote increased collaboration, engagement and sharing of domain knowledge between scientists focused on any aspects of immune-related genomics through the following activities

- to provide a forum for basic science, and clinical, industrial, and educational applications related to immune polymorphism.
- to share and disseminate research findings related to immune polymorphism.
- to assist in the integration of immune polymorphism-related data resources.
- to promote the development of secure open-source, cloud-based technologies that advance the analysis, collection, exchange, and storage of immune-related genomic data.

The goals of SIP are not to replace any of the societies interesting in a specific aspect of immune polymorphisms, but rather to allow for fruitful collaborations between practitioners and researchers in the different domains. We have organized two workshops in Ramat Gan, Israel (Mar, 2018 https://louzouy.wixsite.com/hlakir2018/) and in New Orleans (Oct, 2019 https://immunepolymorphism.tulane.edu/). A plan for this type of article collection was conceived at the first workshop. We plan to continue to work together in these efforts and welcome broad participation from a diverse group of researchers around the globe.

The society is still in its initial stages, further details about the society, its goals, and possible collaborations between SIP and other societies can be obtained from the corresponding authors or the website: immunepolymorphismsociety.org.

## Author Contributions

MM drafted the original. YL, JK, MR, and RM provided comments and feedback. All authors contributed to the article and approved the submitted version.

## Conflict of Interest

The authors declare that the research was conducted in the absence of any commercial or financial relationships that could be construed as a potential conflict of interest.

